# Anti-Melanoma Differentiation-Associated Gene 5 Antibody-Positive Juvenile Dermatomyositis Presenting With Predominant Joint Contractures

**DOI:** 10.31083/RN51324

**Published:** 2026-06-26

**Authors:** Qiyun Liu, Ke Zheng, Ruifang Bai, Qiaoqiao Cui, Lizhen Wang, Jianli Wang, Dan Liu, Juan Wang, Wei Zhang, Xueli Chang

**Affiliations:** ^1^Department of Neurology, The First Hospital of Shanxi Medical University, 030001 Taiyuan, Shanxi, China; ^2^First Clinical Medical College, Shanxi Medical University, 030001 Taiyuan, Shanxi, China

**Keywords:** juvenile dermatomyositis, autoantibodies, contracture, melanoma differentiation-associated gene 5, case reports

## Abstract

**Introduction:**

Anti-melanoma differentiation-associated gene 5 (MDA5) positive juvenile dermatomyositis (JDM) (anti-MDA5+ JDM) is a distinct subtype of JDM characterized by marked clinical heterogeneity. Although cutaneous manifestations and interstitial lung disease (ILD) are well recognized, atypical musculoskeletal presentations may lead to diagnostic delay.

**Clinical Case:**

We report a 16-year-old male with anti-MDA5+ JDM who presented with progressive joint contractures as the predominant manifestation over a 2-year period. The patient also exhibited restricted mouth opening, with a classical dermatomyositis (DM) cutaneous rash absent or only mild, and proximal muscle weakness, mild to moderate. Laboratory evaluation revealed high-titer anti-MDA5 antibodies and a markedly elevated serum immunoglobulin E (IgE) level. Magnetic resonance imaging (MRI) demonstrated periarticular and soft-tissue involvement, and muscle biopsy confirmed pathological features consistent with DM. Treatment with systemic glucocorticoids in combination with methotrexate resulted in substantial improvement in joint mobility, with good tolerability during follow-up.

**Conclusions:**

Progressive contractures can occasionally become the predominant presenting manifestation in anti-MDA5+ JDM and contribute to diagnostic delay. This case underscores the importance of early evaluation for idiopathic inflammatory myopathies (IIM), including myositis-specific antibody (MSA) testing and muscle biopsy, in adolescents with unexplained progressive joint contractures.

## 1. Introduction

Juvenile dermatomyositis (JDM) is a rare systemic autoimmune idiopathic inflammatory myopathies (IIM) characterized by non-purulent inflammation of skeletal muscle and skin, and may involve multiple organ systems [1]. The clinical phenotype of JDM is highly heterogeneous. It is significantly influenced by myositis-specific antibodies (MSAs), which have become a crucial basis for disease stratification, prognosis assessment, and treatment decisions. Among MSAs, anti-melanoma differentiation-associated gene 5 (MDA5) antibodies define a unique JDM subtype that is often associated with characteristic cutaneous manifestations, arthritis, and a high risk of interstitial lung disease (ILD), while muscle weakness and elevated creatine kinase (CK) may be relatively mild [2,3]. In anti-MDA5+ JDM, joint involvement usually presents as inflammatory arthritis affecting the small joints of the hands. Although contractures may occur in anti-MDA5+ JDM, cases in which severe progressive contractures become the predominant presenting manifestation remain insufficiently characterized.

Here, we describe a male adolescent with anti-MDA5+ JDM whose major clinical problem over the disease course was progressive and severe joint contractures, without ILD or persistently elevated CK. This case highlights the diagnostic challenges posed by contracture-dominant musculoskeletal involvement in anti-MDA5+ JDM and suggests that prolonged untreated periarticular inflammation may contribute to severe functional contractures.

## 2. Case Report

A 16-year-old male was admitted to the hospital due to a progressive decline in joint mobility that had persisted for two years. Initially, the condition involved bilateral elbows, manifesting as pain and limited extension. Over time, joint involvement extended to the wrists and metacarpophalangeal (MCP) joints. Subsequently, fixed flexion deformities occurred in the elbow, wrist, and MCP joints, leading to impaired hand function. Concurrently, he developed restricted mouth opening, suggesting involvement of the temporomandibular joints and/or masticatory muscles. During the disease course, the patient reported mild fatigue and reduced exercise tolerance. No typical heliotrope rash or well-demarcated Gottron papules were observed.

**Fig. 1.**
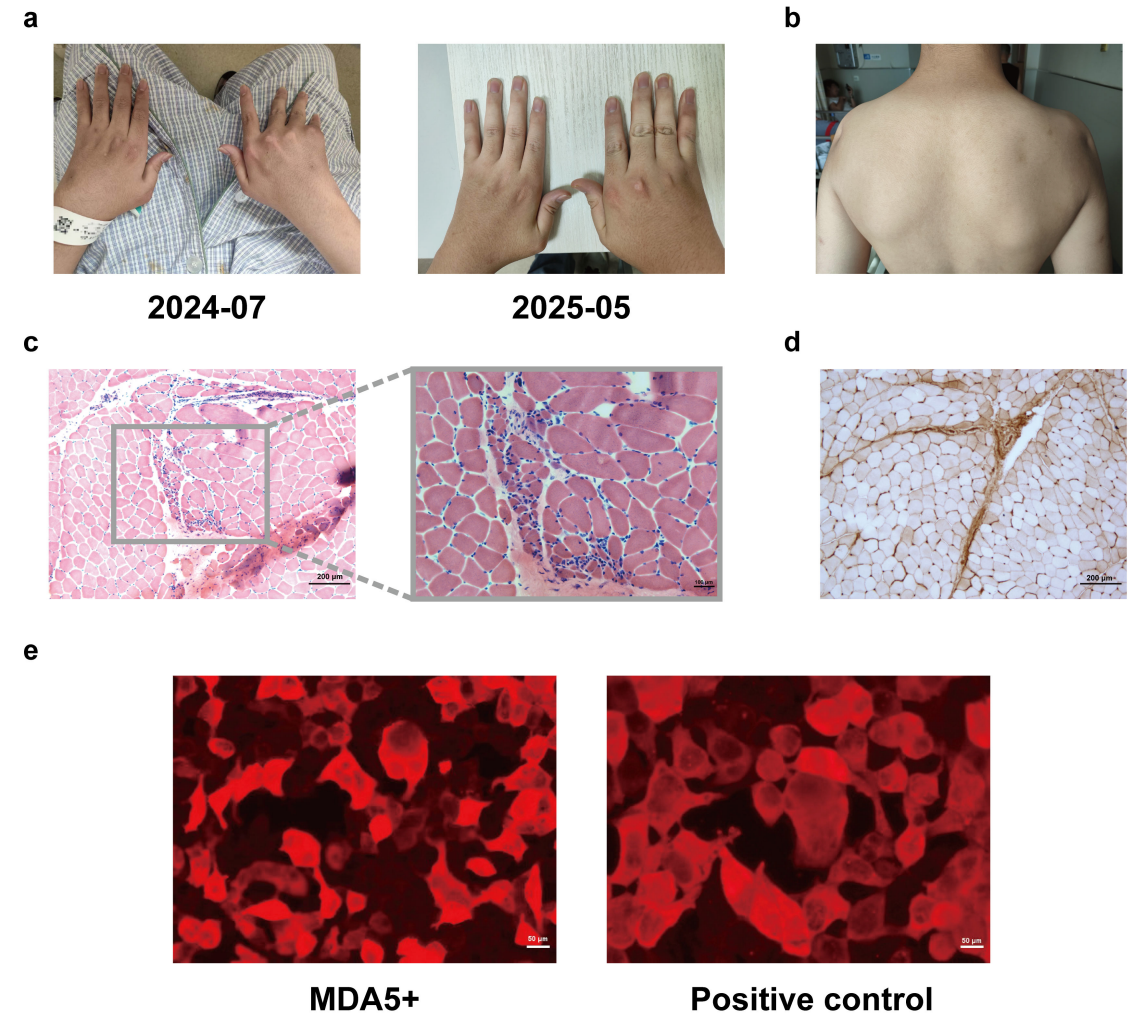
**Clinical features, muscle histopathology, and myositis-specific antibody findings in a patient with anti-melanoma differentiation-associated gene 5 (MDA5)-positive juvenile dermatomyositis (JDM) (anti-MDA5+ JDM)**. (a) Fixed flexion deformities of the metacarpophalangeal (MCP) joints at presentation (July 2024), with limited finger extension, and partial improvement in hand posture after treatment (May 2025). (b) Marked proximal muscle atrophy of the upper limbs. (c) Hematoxylin and eosin–stained muscle biopsy section showing inflammatory cell infiltration in the endomysium and perimysium, with variation in muscle fiber size; the boxed area highlights perifascicular atrophy at higher magnification (scale bar: left, 200 μm; right, 100 μm). (d) Immunohistochemical staining demonstrating diffuse overexpression of major histocompatibility complex (MHC) class I on muscle fibers (scale bar: 200 μm). (e) Cell-based indirect immunofluorescence assay revealing strong cytoplasmic staining consistent with anti-MDA5 antibody positivity (left), with a positive control shown for comparison (right) (scale bar: 50 μm).

On admission, both MCP joints showed fixed flexion deformities and could not be actively or passively extended beyond an approximately 120° flexed position (Fig. 1a). Marked proximal muscle atrophy of the upper limbs was evident (Fig. 1b). Upon mouth opening, the maximum interincisal distance of the patient was measured to be approximately 30 mm. Neurological examination revealed mild to moderate proximal muscle weakness, graded using the Medical Research Council (MRC) scale as follows: proximal upper limbs 3/5, distal upper limbs 4/5, and both proximal and distal lower limbs 4/5. Muscle function was assessed using the Childhood Myositis Assessment Scale (CMAS; range 0–52, with higher scores indicating better function) [4]. At baseline, the CMAS score was 45/52.

Laboratory investigations demonstrated a markedly elevated serum immunoglobulin E (IgE) level of 3788 kU/L (reference range <100 kU/L). Serum CK and lactate dehydrogenase (LDH) levels were within the reference range at admission; however, a transient elevation of CK (400–500 U/L) had been documented earlier in the disease course.

Electromyography (EMG) demonstrated reduced compound muscle action potential amplitudes in the bilateral ulnar nerves and the left median nerve, along with shortened motor unit potential durations in the right deltoid muscle and the right extensor digitorum muscles. On hospital day four, a left biceps brachii muscle biopsy was performed. The findings revealed inflammatory cell infiltration in the endomysium, perimysium, and perivascular regions, along with marked variation in muscle fiber size, characteristic perifascicular atrophy, and major histocompatibility complex (MHC) class I positivity (Fig. 1c,d). These findings are consistent with dermatomyositis (DM). MSA testing was performed using a cell-based indirect immunofluorescence assay panel. Anti-MDA5 antibodies were strongly positive at a dilution of 1:300 (Fig. 1e), whereas anti-nuclear matrix protein 2 (NXP2) and the other tested MSAs were negative.

**Fig. 2.**
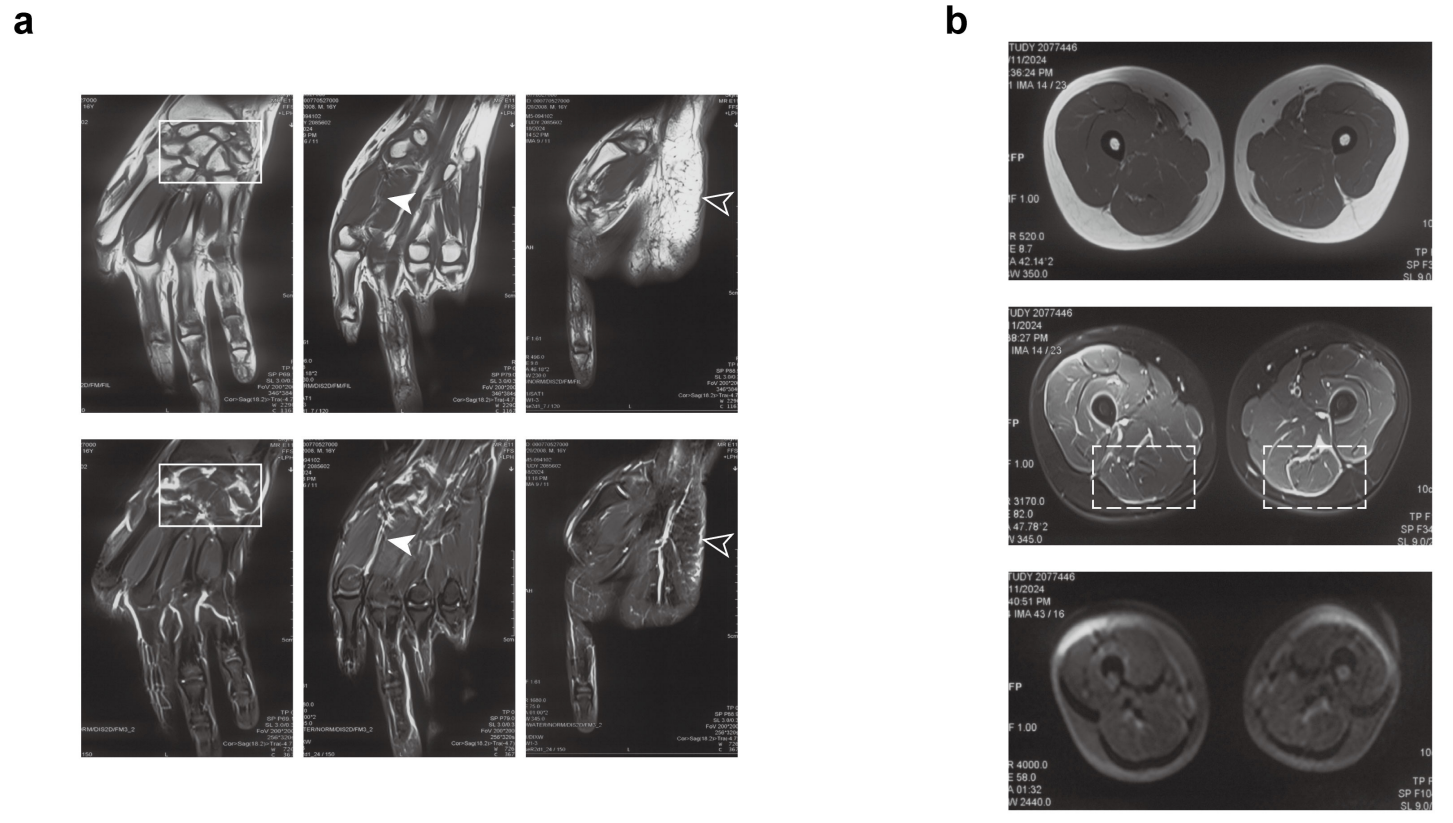
**Magnetic resonance imaging (MRI) findings demonstrating musculoskeletal involvement**. (a) MRI of the hands and wrists showing ulnar deviation of both wrists, synovial hyperplasia, multifocal bone marrow edema of the carpal bones (boxed areas), peritendinous fluid surrounding the flexor and extensor tendons (solid arrowheads), and soft tissue edema around the wrist joints (open arrowheads). (b) Axial MRI of the lower limbs demonstrating hyperintense signal changes in the fascia and adjacent muscle compartments of the bilateral biceps femoris muscles on fluid-sensitive sequences (dashed boxes), indicating myofascial inflammation; corresponding images without signal abnormalities on non–fluid-sensitive sequences are shown for comparison.

An upper limb joint ultrasound was also performed during hospitalization. It demonstrated synovial hypertrophy of the left wrist joint with bony erosion, synovial hypertrophy of the right wrist joint, synovial hypertrophy involving the right MCP and interphalangeal joints, and effusion of the left elbow joint. Magnetic resonance imaging (MRI) of the hands and wrists demonstrated ulnar deviation of both wrists, synovial hyperplasia, multifocal bone marrow edema of the carpal bones, peritendinous fluid surrounding the flexor and extensor tendons, and soft tissue edema around the wrist joints (Fig. 2a). MRI of the lower limbs revealed fascial edema involving the bilateral biceps femoris muscles (Fig. 2b). Cardiac evaluation, pulmonary function testing, and chest computed tomography (CT) revealed no abnormalities, and there was no evidence of ILD. Although classic cutaneous findings were absent or subtle and serum CK was not persistently elevated, the combination of objective proximal muscle weakness, myopathic EMG abnormalities, MRI evidence of inflammatory soft-tissue/fascial involvement, inflammatory muscle biopsy findings with perifascicular atrophy and MHC class I overexpression, and high-titer anti-MDA5 antibodies supported the diagnosis of anti-MDA5+ JDM.

On hospital day nine, the patient was started on intravenous methylprednisolone 80 mg/day (1.33 mg/kg/day) for 7 days. By the time of discharge, symptoms of restricted joint mobility had not shown further progression, and the clinical condition was stable. Accordingly, glucocorticoid therapy was transitioned to oral prednisone at 50 mg/day in combination with methotrexate. Methotrexate was started at 10 mg/week, administered orally, with folic acid supplementation. Prednisone was gradually tapered every two weeks to a maintenance dose of 5 mg/day, while methotrexate was administered as a steroid-sparing immunosuppressive agent. At the 1-month follow-up, the patient showed no further deterioration in joint contracture symptoms. After 4 months of treatment, the patient felt that the stiffness in both hands had improved compared to before. At the 10-month follow-up, while receiving a stable low dose of prednisone (5 mg/day), the contractures of the wrist and MCP joints showed significant improvement, enabling the patient to place both palms flat on the table. The CMAS score increased to 47/52. At follow-up, pulmonary status was monitored through interval clinical assessment for respiratory symptoms and signs, together with ongoing consideration of repeat pulmonary function testing and chest imaging if clinically indicated. Because the patient remained free of respiratory symptoms and had no baseline evidence of ILD on pulmonary function testing and chest CT, repeat chest CT was not performed during the 10-month follow-up period.

This case report was prepared in accordance with the CARE guidelines. The completed CARE checklist is provided as **Supplementary Material**.

## 3. Discussion

Anti-MDA5+ JDM is a distinct subtype of JDM that is associated with a higher frequency of joint involvement, commonly presenting as arthritis affecting the small joints [2,5]. In addition, a large North American cohort study by Mamyrova et al. [6] reported contractures in up to 45.7% of patients, indicating that contractures as a form of joint involvement are not uncommon in this subtype. In this context, the striking feature of this case was that severe, progressive, and function-limiting contractures became the predominant presenting manifestation over a prolonged course, thereby obscuring the underlying IIM and contributing to diagnostic delay.

Joint contractures are reported more frequently in anti-NXP2+ JDM; this difference is commonly attributed to the higher prevalence of calcinosis in the NXP2 subtype [7,8]. Notably, calcinosis was not observed clinically in our patient. Instead, musculoskeletal imaging supported active periarticular inflammation: ultrasound demonstrated synovial abnormalities involving the wrists and hand joints, whereas MRI showed synovial hyperplasia, peritendinous fluid, soft-tissue edema around the wrists, and fascial edema in the lower limbs. Together, these findings suggest that chronic periarticular inflammation and secondary fibrosis may have contributed to the development of contractures. Therefore, the severe contractures observed in this patient may not represent an isolated disease-specific phenotype, but rather a late and functionally significant consequence of prolonged untreated articular and periarticular inflammation.

In addition to joint contractures, the present patient also exhibited restricted mouth opening, defined as a maximal interincisal distance of less than 40 mm, indicating reduced mandibular mobility [9]. Orofacial involvement, while not traditionally emphasized, represents a clinically relevant manifestation of IIM. Previous studies have shown that patients with IIM have a significantly higher prevalence of impaired mandibular mobility and reduced mouth opening compared with healthy controls, findings that are most commonly attributed to involvement of the masticatory muscles [10]. Taken together, these musculoskeletal and orofacial findings indicate that pediatric IIM may initially manifest as functional impairment rather than overt muscle weakness or typical skin features. Accordingly, JDM should be considered in the differential diagnosis of adolescents presenting with unexplained progressive joint stiffness, joint contractures, or restricted mandibular movement, even in the absence of a characteristic skin rash or prominent proximal muscle weakness.

The differential diagnosis in this case deserves particular emphasis because the disease evolved slowly over approximately 2 years and the muscle manifestations were relatively subtle. From an articular perspective, chronic inflammatory arthritis such as juvenile idiopathic arthritis could be considered [11], especially in view of the synovial abnormalities involving the elbows, wrists, and MCP joints. However, this explanation alone could not account for the proximal muscle weakness, upper-limb muscle atrophy, myopathic electromyographic changes, fascial inflammation on MRI, and the characteristic muscle biopsy findings. In addition, inherited disorders associated with early contractures and relatively mild weakness, such as collagen VI-related myopathies and Emery–Dreifuss muscular dystrophy, were important considerations [12,13]. Nevertheless, the presence of inflammatory muscle pathology with perifascicular atrophy and MHC class I overexpression, together with high-titer anti-MDA5 antibodies and clinical improvement after immunosuppressive treatment, strongly supported an acquired IIM rather than a hereditary dystrophic process. Therefore, the overall clinicopathological picture was most consistent with anti-MDA5+ JDM.

The markedly elevated serum IgE level observed in the current patient is of particular interest. While JDM linked with hyper-IgE syndrome has been documented before, these cases typically involve recurrent infections [14], which were not present in our patient, who also had no history of asthma, eczema, or allergic rhinitis. However, other common causes of extreme IgE elevation, including parasitic infection and additional allergic conditions, were not systematically evaluated in the present case. Therefore, we cannot determine whether the elevated IgE was related to the underlying inflammatory disease or represented an incidental immunologic abnormality.

At present, there is no standardized treatment strategy for anti-MDA5+ JDM, and therapeutic approaches remain largely empirical. Intensive combination immunosuppressive regimens have been associated with improved survival in patients with rapidly progressive ILD, although at the expense of a higher risk of serious infections [15]. In contrast, the present patient showed no evidence of severe visceral involvement and was therefore managed with glucocorticoids in combination with methotrexate. During follow-up, substantial improvement in joint contractures was observed, with good treatment tolerability. The modest improvement in CMAS score over the 10-month follow-up period suggests stabilization with mild functional improvement [16]. This clinical course suggests that glucocorticoids combined with methotrexate may be an effective and relatively well-tolerated therapeutic option for patients with anti-MDA5+ JDM who present predominantly with musculoskeletal involvement and no major organ complications.

The lack of repeat radiological follow-up represents an important limitation of the present case report. Although no evidence of ILD was identified at presentation, pulmonary surveillance remains important in anti-MDA5+ JDM because lung involvement may emerge during the disease course and may not always be heralded by prominent respiratory symptoms [2,17]. In our patient, baseline pulmonary function testing and chest CT were normal, and no respiratory symptoms developed during follow-up. For this reason, we adopted continued clinical respiratory surveillance, with repeat pulmonary function testing and chest imaging to be considered if respiratory symptoms, examination abnormalities, or other clinical concerns arose. Nevertheless, because a repeat chest CT was not performed, interval subclinical pulmonary changes cannot be completely excluded. Another limitation of this case is that anti-MDA5 positivity was identified by a cell-based indirect immunofluorescence assay alone. Confirmatory testing using an additional method, such as immunoblotting, ELISA, or immunoprecipitation, was not performed. Therefore, although the serological result was supportive in the context of the overall clinicopathological findings, the possibility of assay-related false positivity or cross-reactivity cannot be completely excluded.

## 4. Conclusions

This case highlights the diagnostic challenges posed by a contracture-dominant musculoskeletal presentation in anti-MDA5+ JDM. Rather than the mere presence of contractures, the notable feature of this case is that progressive contractures represented the predominant clinical manifestation and delayed recognition of the underlying IIM. Early recognition, supported by MSA testing and muscle biopsy, is critical for establishing the diagnosis and guiding management.

## Data Availability

Study data are available from the corresponding author upon request.

## Abbreviations

CK, creatine kinase; CMAS, Childhood Myositis Assessment Scale; DM, dermatomyositis; EMG, electromyography; IgE, immunoglobulin E; IIM, idiopathic inflammatory myopathies; ILD, interstitial lung disease; JDM, juvenile dermatomyositis; LDH, lactate dehydrogenase; MCP, metacarpophalangeal; MDA5, melanoma differentiation-associated gene 5; MHC, major histocompatibility complex; MRC, Medical Research Council; MRI, magnetic resonance imaging; MSA, myositis-specific antibody.

## References

[1] Papadopoulou C, Chew C, Wilkinson MGL, McCann L, Wedderburn LR (2023). Juvenile idiopathic inflammatory myositis: an update on pathophysiology and clinical care. Nature Reviews Rheumatology.

[2] Tansley SL, Betteridge ZE, Gunawardena H, Jacques TS, Owens CM, Pilkington C (2014). Anti-MDA5 autoantibodies in juvenile dermatomyositis identify a distinct clinical phenotype: a prospective cohort study. Arthritis Research & Therapy.

[3] Tansley SL, Simou S, Shaddick G, Betteridge ZE, Almeida B, Gunawardena H (2017). Autoantibodies in juvenile-onset myositis: Their diagnostic value and associated clinical phenotype in a large UK cohort. Journal of Autoimmunity.

[4] Lovell DJ, Lindsley CB, Rennebohm RM, Ballinger SH, Bowyer SL, Giannini EH (1999). Development of validated disease activity and damage indices for the juvenile idiopathic inflammatory myopathies. II. The Childhood Myositis Assessment Scale (CMAS): a quantitative tool for the evaluation of muscle function. Arthritis and Rheumatism.

[5] Horn S, Minden K, Speth F, Schwarz T, Dressler F, Grösch N (2022). Myositis-specific autoantibodies and their associated phenotypes in juvenile dermatomyositis: data from a German cohort. Clinical and Experimental Rheumatology.

[6] Mamyrova G, Kishi T, Shi M, Targoff IN, Huber AM, Curiel RV (2021). Anti-MDA5 autoantibodies associated with juvenile dermatomyositis constitute a distinct phenotype in North America. Rheumatology (Oxford, England).

[7] Miura E, Taneda T, Umeda Y, Umeda M, Oyake M, Matsushita T (2024). Juvenile-onset anti-nuclear matrix protein 2 (NXP-2) antibody-positive dermatomyositis with joint contractures before manifestation of myositis: a case report. Clinical Neurology.

[8] Ichimura Y, Konishi R, Shobo M, Inoue S, Okune M, Maeda A (2022). Anti-nuclear matrix protein 2 antibody-positive inflammatory myopathies represent extensive myositis without dermatomyositis-specific rash. Rheumatology (Oxford, England).

[9] Crincoli V, Cannavale M, Cazzolla AP, Dioguardi M, Piancino MG, Di Comite M (2021). Temporomandibular Disorders and Oral Features in Idiopathic Inflammatory Myopathies (IIMs) Patients: An Observational Study. International Journal of Medical Sciences.

[10] Savioli C, Silva CAA, Fabri GMC, Kozu K, Campos LMA, Bonfá E (2010). Gingival capillary changes and oral motor weakness in juvenile dermatomyositis. Rheumatology (Oxford, England).

[11] Ventura-Ríos L, Faugier E, Barzola L, De la Cruz-Becerra LB, Sánchez-Bringas G, García AR (2018). Reliability of ultrasonography to detect inflammatory lesions and structural damage in juvenile idiopathic arthritis. Pediatric Rheumatology Online Journal.

[12] Bönnemann CG (2011). The collagen VI-related myopathies: muscle meets its matrix. Nature Reviews. Neurology.

[13] Ben Yaou R, Leturcq F, Bonne G, Adam MP, Bick S, Mirzaa GM, Pagon RA, Wallace SE, Amemiya A (1993–2026). Emery-Dreifuss muscular dystrophy. GeneReviews®.

[14] Saikia B, Aneja H, Jain J, Puliyel JM (2013). Hyperimmunoglobulin E syndrome with juvenile dermatomyositis and calcinosis. Clinical Rheumatology.

[15] Tsuji H, Nakashima R, Hosono Y, Imura Y, Yagita M, Yoshifuji H (2020). Multicenter Prospective Study of the Efficacy and Safety of Combined Immunosuppressive Therapy With High-Dose Glucocorticoid, Tacrolimus, and Cyclophosphamide in Interstitial Lung Diseases Accompanied by Anti-Melanoma Differentiation-Associated Gene 5-Positive Dermatomyositis. Arthritis & Rheumatology (Hoboken, N.J.).

[16] Huber AM, Feldman BM, Rennebohm RM, Hicks JE, Lindsley CB, Perez MD (2004). Validation and clinical significance of the Childhood Myositis Assessment Scale for assessment of muscle function in the juvenile idiopathic inflammatory myopathies. Arthritis and Rheumatism.

[17] Hallowell RW, Paik JJ (2022). Myositis-associated interstitial lung disease: a comprehensive approach to diagnosis and management. Clinical and Experimental Rheumatology.

